# Altered cerebellar–insular–parietal–cingular subnetwork in adolescents in the earliest stages of anorexia nervosa: a network–based statistic analysis

**DOI:** 10.1038/s41398-018-0173-z

**Published:** 2018-07-06

**Authors:** Santino Gaudio, Gaia Olivo, Bruno Beomonte Zobel, Helgi B. Schiöth

**Affiliations:** 1 Department of Neuroscience, Functional Pharmacology, Uppsala University, BMC, Box 593, 751 24 Uppsala, Sweden; 2 Eating Disorders Centre “La Cura del Girasole” ONLUS, Via Gregorio VII, 186/B, 00165 Rome, Italy; 3 Area of Diagnostic Imaging, Departmental Faculty of Medicine and Surgery, Università “Campus Bio-Medico di Roma”, via Alvaro del Portillo, 200, 00133 Rome, Italy

## Abstract

To date, few functional magnetic resonance imaging (fMRI) studies have explored resting-state functional connectivity (RSFC) in long-lasting anorexia nervosa (AN) patients via graph analysis. The aim of the present study is to investigate, via a graph approach (i.e., the network-based statistic), RSFC in a sample of adolescents at the earliest stages of AN (i.e., AN duration less than 6 months). Resting-state fMRI data was obtained from 15 treatment-naive female adolescents with AN restrictive type (AN-r) in its earliest stages and 15 age-matched healthy female controls. A network-based statistic analysis was used to isolate networks of interconnected nodes that differ between the two groups. Group comparison showed a decreased connectivity in a sub-network of connections encompassing the left and right rostral ACC, left paracentral lobule, left cerebellum (10th sub-division), left posterior insula, left medial fronto-orbital gyrus, and right superior occipital gyrus in AN patients. Results were not associated to alterations in intranodal or global connectivity. No sub-networks with an increased connectivity were identified in AN patients. Our findings suggest that RSFC may be specifically affected at the earliest stages of AN. Considering that the altered sub-network comprises areas mainly involved in somatosensory and interoceptive information and processing and in emotional processes, it could sustain abnormal integration of somatosensory and homeostatic signals, which may explain body image disturbances in AN. Further studies with larger samples and longitudinal designs are needed to confirm our findings and better understand the role and consequences of such functional alterations in AN.

## Introduction

Anorexia nervosa (AN) mainly affects adolescent girls and young women and it is characterized by extremely low body weight, intense fear of weight gain, body image distortion, and food refusal^1^. Although AN is a severe mental disorder with the highest mortality rate of psychiatric disorders^2^, its etiology still remains insufficiently understood and there is still not a widely accepted treatment strategy^3,4^. To date, several factors are considered involved in AN pathogenesis: genetic, neurobiological, and psychosocial^3,4^. In particular, novel neuroimaging tools have provided new and helpful insights into the understanding of structural and functional neural correlates of AN. Structural neuroimaging studies have shown that acutely underweight AN patients have gray (GM) and white matter (WM) loss^5^, that seem to be reversible after weight restoration^6^. Functional neuroimaging studies have revealed that, in response to specific tasks, AN patients have specific functional alterations mainly related to altered eating and reward processing and body image disturbances (for a review, see refs. ^3,7^, respectively).

In the last years, a growing interest has been focused on the functional connectivity of the brain at rest in AN^8^. Resting-state functional MRI (fMRI)^9^ investigates functional connectivity, by recognizing temporal correlations in Blood-Oxygen Level-Dependent signal between spatially distinct brain areas^10^. The resting-state fMRI approach is useful for clinical application, as it has a quite short scanning time and it needs modest compliance from the participant. Resting-state fMRI data can be analyzed using a variety of approaches. The most commonly used are independent component analysis (ICA) and seed-based approaches^11^. The ICA approach allows researchers to examine well-recognized intrinsic neural networks without the need for a priori hypothesis^12–14^. The seed-based approach is driven by an a priori hypothesis and it investigates functional connectivity between a predefined seed region and other brain regions^11^. Graph analysis and effective connectivity are other novel approaches to study brain functional connectivity. In particular, graph analysis considers the brain as a complex network consisting of nodes connected by edges, where nodes are selected brain areas distributed across the whole brain and edges are the functional relationship between nodes^15^. In this framework, a number of measures relative to the global and local properties of brain connections can be measured with appropriate statistical approaches, reflective of the cost-effectiveness of the network. A new graph statistical approach is the network-based statistics (NBS)^16^. It is a validated statistical method to address the multiple comparisons problem when analyzing connectivity graphs and allows to identify sub-networks and connections showing between-group differences (for details, see ref. ^16^).

Resting-state fMRI studies on AN patients have adopted all the above-mentioned approaches, also using different a priori hypotheses (for a review, see ref. ^8^). In particular, altered functional connectivity has been found in different networks: the default mode, visual, somatosensory, fronto-parietal, executive control, and salience networks in participants with current or past AN in ICA-based studies^17–22^. Functional connectivity differences have also been found in the dorsal anterior cingulate cortex (ACC)^23^, the thalamus^24^, and the right inferior frontal gyrus^25^ between AN patients and controls in seed-based studies. Finally, graph analysis studies showed functional alterations mainly involving the insula^26,27^ and the thalamus^27,28^. In particular, one study adopted the NBS approach and showed that AN patients had a reduced connectivity in a subnetwork including the posterior insula, putamen thalamus, amygdala, and fusiform gyrus^26^. On the whole, the above-mentioned alterations may reflect cognitive control processing and visual and homeostatic integration impairments, and seem to be related to the main symptoms of AN: cognitive inflexibility and body image distortion^8^.

Previous graph analysis studies enrolled AN patients with a long disease duration and one or more confounding factors (e.g., psychiatric comorbidity, pharmacological treatment)^26–28^. To date, as malnutrition and dehydration may affect neuroimaging findings^3^, a key open question in the field remains on whether structural and functional brain alterations are the cause or the consequence of AN^3^.

The aim of the present study is to investigate, via the NBS statistical approach, whole-brain resting-state functional connectivity (RSFC) in a sample of treatment naive subjects with AN at its earliest stages of the disease in order to minimize the role of brain atrophy, which con occur in AN^5^ and may affect neuroimaging findings^3^, and also excluding other possible confounding factors (i.e., pharmacological treatment and psychiatric comorbidity). It has been also explored whether functional connectivity changes identified with the NBS can be explained by regional connectivity differences to replicate Ehrlich and colleagues^26^. In addition, selected global and local graph measures were explored within functional connectivity alterations identified with the NBS. Finally, correlation analyses between network connectivity measures and selected clinical variables (i.e., BMI, AN duration, and five selected eating disorder inventory-II subscales, Beck depression inventory-II, and state-trait anxiety inventory-trait scores) were performed. We hypothesize that altered RSFC may be present since the earliest stages of AN and could also involve networks/areas identified in previous graph analysis studies on long-lasting AN patients^26–28^.

## Methods

### Participants

A sum of 36 right-handed female adolescents (19 outpatients with AN-restrictive type (AN-r) and 17 healthy subjects) were recruited and scanned. Four AN-r outpatients and two controls were excluded: two AN-r patients were excluded due to MRI artefacts that significantly decreased the image quality, secondary to orthodontic braces, two AN-r patients were excluded due to scans corruption, and two controls were excluded for excessive head movement (for details on this last point, see the preprocessing paragraph). Except one AN patient and one control subject subsequently recruited, the recruited sample overlaps with two previous published paper using a whole-brain ICA-based resting-state approach^20^ and a diffusion tensor imaging analysis^29^, respectively. The study sample was composed of 15 adolescent females with AN-r and 15 age-matched healthy adolescent female controls. The AN-r subjects were recruited from a non-profit outpatient treatment center for eating disorders (i.e., “La cura del girasole ONLUS”) in Rome (Italy). The inclusion criteria for the clinical sample were: 13–18 years of age; a diagnosis of AN-r in accordance with DSM-IV-TR criteria;^30^ duration of AN-r less than 6 months at the time of scanning; and right-handedness. The exclusion criteria were a previous history of other eating disorders; the presence of a binge eating/purging type of AN; the presence of other current or previous psychiatric disorders (DSM-IV-TR); current or former use of psychoactive medication; concomitant medical diseases; history of neurological diseases or head trauma; and the presence of any absolute contraindication for MRI. All patients were under diagnostic evaluation for AN (all procedures of the study were completed within 1 week after the first clinical interview).

The control sample was recruited in a high school of the same geographic area of the patients sample. The inclusion criteria for the control group were: 13–18 years of age and right-handedness. Exclusion criteria included a history of eating disorders or comorbid psychiatric disorders (as defined by the DSM-IV-TR); any history of treatment with psychoactive mediation; concomitant medical diseases; history of neurological problems or head trauma; and the presence of any absolute contraindication for MRI.

At the time of scanning, all participants of both groups had received similar schooling.

The present study was conducted according to the declaration of Helsinki and it was approved by the “La cura del girasole” ONLUS institutional review board. Written informed consent was directly obtained from participants and parents of those participants younger than 18 and from those participants who had reached the age of 18.

### Clinical assessment and tools

All participants underwent the same psychopathological assessment. Diagnosis of AN and other current or past EDs was made by a clinical interview that was performed according to the eating disorders section of the Structured Clinical Interview for DSM-IV^31^. Diagnosis of past or current other Axis I disorders was made in accordance with DSM-IV-TR criteria^30^ by a comprehensive clinical interview. Personality disorders were assessed in patients older than 16 years of age^32^ by using The Italian version of the Structured Clinical Interview for Axis II Disorders (SCID II)^33^. In addition, all participants were also asked to complete the Italian version of the Eating Disorder Inventory (EDI-2)^34^ for drive for thinness, body dissatisfaction, interoceptive awareness, perfectionism, and bulimia; the Italian version of the Beck Depression Inventory-II (BDI-II);^35^ and the Italian version of the State-Trait Anxiety Inventory–form Y (STAI-Y)^36^ for trait anxiety.

All interviews and self-report questionnaires were carried out by the first author, who has more than 10 years of expertise in eating disorders in children and adolescents and in child and adolescent psychiatry and who was specifically trained to use the diagnostic tools applied.

### MRI acquisition

Structural and functional scans were acquired with a Siemens 1.5-Tesla MAGNETOM Avanto (Siemens, Erlangen, Germany) using a 12-element designed Head Matrix coil. Structural images were acquired using a 3D-MPRAGE T1-weighted sequence (TR = 1900 ms; TE = 3.37 ms; flip angle: 15°; slice thickness = 1.3 mm; no gap between slices). The resting-state fMRI was acquired using a T2^*^-weighed EPI sequence (TR = 3560 ms; TE = 50 ms; flip angle: 90°; slice thickness = 3 mm; number of slices = 36; no inter-slice spacing) and 80 volumes were registered.

### Pre-processing

All pre-processing was carried out using DPARSFA (Data Processing Assistant for Resting-State fMRI Advanced; http://rfmri.org/DPARSF). Slice-timing correction was applied to the functional volumes, then they were realigned to correct for head movements. A threshold of 3 mm was set as an exclusion criterion for excessive head movement; two controls had moved more than 3 mm and were thus excluded from further analyses. For further analyses 15 patients and 15 controls were retained.

Structural volumes were first coregistered to the functional images, then segmented using DARTEL (Diffeomorphic Anatomical Registration Through Exponentiated Lie Algebra). The volumes were segmented into GM, WM, and cerebro-spinal fluid (CSF) probability maps and a sample-specific local template was created. The template was normalized to the MNI space. The resulting deformations were applied to the native space segmented GM images of each subject. The GM maps were resampled to a 2 × 2 × 2 mm^3^ voxel size, modulated to preserve the amount of GM, and smoothed using a 8 mm FWHM isotropic Gaussian kernel. The normalized images were visually inspected to ensure good quality of the normalization.

Functional images were denoised via the removal of WM and CSF effects. Band-pass filtering (0.01–0.08 Hz) was performed to avoid artefacts due to physiological noise (such as breathing or heart pulse-related movements), then the functional images were normalized to the MNI space using the normalization parameters and flow-fields deriving from the segmentation procedure of the corresponding structural images and smoothed with a 4 FHWM Gaussian kernel.

### Voxel-based morphometry (VBM)

Considering that GM atrophy may affect functional connectivity analyses^37^ and it is well known in AN^5^, a VBM analysis was performed using SPM12 to explore structural brain differences between groups. An independent sample analysis was carried out to test for differences in local GM volume between patients and controls. The threshold for significance was set at *p* < 0.05, corrected for family-wise error (FWE) rate. The total GM, WM, and CSF volume were also extracted and tested for differences between patients and controls, with a significance threshold of *p* < 0.05, corrected for multiple comparisons.

### Regions of interest (ROIs) definition

Time-course extraction was carried out on 128 ROIs, using DPARSFA. For time-course extraction, the automated anatomical labeling (AAL) atlas^38^ ROIs, implemented in DPARSFA, were selected. Out of the 116 included in the AAL atlas, 112 ROIs were selected; we did not include the insula (right and left) and the ACC (right and left). Instead, following the approach used in ref. ^27^, we split the left and right insulae and the ACC in more sub-regions, to account for the anatomical^39^, biochemical^40^, and functional^41^ specificity of their different sub-regions, by drawing additional sphere-shaped ROIs with a radius of 5 mm. Specifically, the insula was sub-divided in three sub-regions for each hemisphere, as identified by previous cluster-analyses:^42^ ventral-anterior (MNI coordinates: −33, 13, −7 and 32, 10, −6, left and right), dorsal-anterior (MNI coordinates: −38, 6, 2 and 35, 7, 3, left and right), and posterior insula (MNI coordinates: −38, −6, 5 and 35, −11, 6, left and right)^42^. The ACC was subdivided in five sub-regions for each hemisphere^43^, as identified by functional connectivity analyses:^44^ caudal ACC (MNI coordinates: ±5, −10, 47), dorsal ACC (MNI coordinates: ±5, 14, 42), and rostral ACC (MNI coordinates: ±5, 34, 28)^43–45^. Thus, time-courses were extracted by a total of 128 ROIs (112 from the AAL atlas, 6 insular ROIs, and 10 ACC ROIs), representative of the whole brain. For a detailed list of ROIs coordination and labels, please see Supplementary Material, Table S1.

### Network-based statistics

The brain works as an interconnected network, which can be represented as graph consisting of nodes and edges. Nodes represent different specialized regions, while edges represent communication pathways or connections^37^. Graph analysis can effectively describe the topological properties of a network at a local and global level; however, graph analysis involves a huge number of multiple comparisons, as it operates a correction for FWE rate independently for each link in the network (link-based statistics). Other approaches, such as the NBS approach^16^, can yield greater power than what is achievable with a graph analysis^16^.

The NBS approach is based on cluster statistics, and consists of three steps^16^. As a first step, NBS identifies node-to-node connections (links) that surpass a given threshold. In the next step, connected structures (networks) are identified within these supra-threshold links. In the last step, a permutation testing is carried out to assign a *p*-value (controlled for the FWE) to each network based on its size. NBS allows identifying connections and networks associated with an experimental effect or a between-group difference. The NBS does not provide detailed information about the connectivity characteristics of the individual nodes in the network.

The NBS was performed using Network-Based Statistics Toolbox (https://sites.google.com/site/bctnet/comparison/nbs). The primary threshold (i.e., the test statistic threshold), which adjusts the extremity of deviation in a connection between groups^16^, was set at different values in the range between 2.0 and 4.0^26^. The network threshold parameter influences the extent of the returned network; however, no standard value has been established, and the threshold selection is arbitrary^16^. Therefore, experimenting with a range of thresholds is recommended (for details on primary thresholding process, see Zalesky and colleagues^16^). The significance threshold for the analysis was set at *p* < 0.05 corrected for multiple comparisons with a FWE rate approach and 10,000 randomizations were performed. The NBS approach is described in depth in Zalesky and colleagues^16^.

### Intranodal homogeneity

Intranodal homogeneity was calculated for the nodes emerged from the NBS using DPARSFA, by estimating Kendall’s coefficient concordance (KCC) for all voxels within each node implicated in the subnetwork (identified by NBS at *t* = 3.7) as a measure of ReHo. Differences between groups in the KCC of each node were tested using Statistical Package for Social Science 24.0 (SPSS). The threshold for significance was set at *p* > 0.007, corrected for multiple comparisons according to Bonferroni (0.05/7 nodes).

Partial correlations between KCC of each node and inter-regional connectivity measured by NBS analysis were assessed by Pearson’s coefficient, controlling for age. Six edges (connections) were tested against KCC of the respective nodes. The threshold for significance was set at *p* > 0.004, corrected for multiple comparisons according to Bonferroni (0.05/(6 edges × 2 nodes each)).

### Graph analysis

Graph analysis was performed with BRAPH toolbox (BRain Analysis using graPH theory)^46^. Seven nodes, which emerged as statistically significant at the NBS (see results), were selected as network: left and right rostral ACC, left medial fronto-orbital gyrus, left paracentral lobule, left 10th lobule of the cerebellum, right superior occipital gyrus, left posterior insula. Weighted, undirected graphs were constructed for each subject. Pearson’s correlation coefficients were computed, and the absolute value of the correlations was considered. Indeed, the weight and the presence of a statistical interaction are more relevant than the sign of the correlation, which could also present difficulties in terms of neurophysiological interpretation^37^. Six global measures and seven local measures were computed (for details, see Supplementary Material). An amount of 10,000 permutations were computed. The significance threshold was set at *p* < 0.008 (0.05/6) for global measures and at *p* < 0.001 (0.05/(7 nodes × 6 measures)) for local measures, to account for multiple testing according to Bonferroni.

### Statistical analysis of clinical measures and correlation analysis

Statistical analysis of clinical data was performed with SPSS. The Shapiro–Wilk test was used to test for normal distribution in demographic and clinical data. The Student’s *t*-test or Mann–Whitney U test was performed, where appropriate, to test for group differences in age, BMI, BDI, five EDI-2 subscales (i.e., drive for thinness, body dissatisfaction, interoceptive awareness, perfectionism, and bulimia), and STAI-trait scores.

Partial correlations between clinical variables and correlation coefficients relative to each of the six connections identified by the NBS (see NBS results) were also assessed by Pearson’s or Spearman’s coefficient where appropriate, controlling for age. Within controls, BMI, BDI, STAI-trait, drive for thinness, body dissatisfaction, interoceptive awareness, perfectionism, bulimia were tested, with a threshold of *p* < 0.0008 (0.05/10 clinical variables/6 connections) corrected for multiple comparisons according to Bonferroni. Within patients, AN duration was tested as well, setting the threshold for significance at *p* < 0.0007 (0.05/11 clinical variables/6 connections).

## Results

### Sample characteristics

Table 1 shows the clinical features of the AN sample and control groups. The two groups had no differences in age. The AN sample showed significantly lower BMI and a significantly higher drive for thinness, body dissatisfaction, interoceptive awareness, perfectionism, BDI, and STAI-Trait. Bulimia subscale score did not significantly differ between groups. AN patients did not meet the DSM-IV-TR criteria for other Axis I or Axis II disorders. AN patients had no previous or current psychopharmacological treatments.

**Table 1:** Demographics and clinical data and GM, WM, and CSF total volumes

	AN-r (*N* = 15)		HC (*N* = 15)			
	Mean	(SD)	Mean	(SD)	Statistic	*p*
Age (years)	15.7	(1.7)	16.1	(1.4)	0.727^a^	<0.473
BMI	16.1	(1.2)	21.6	(2.4)	7.966^a^	<0.001
Lifetime lowest BMI	16	(1.3)	–	–	–	–
Age of onset (years)	15.2	(1.6)	–	–	–	–
Disease duration (months)	4.0	(1.8)	–	–	–	–
Beck Depression Inventory	31	(12)	5	(3)	223.500^b^	<0.001
State Trait Anxiety Inventory-trait	53	(14)	30	(5)	214.000^b^	<0.001
Drive for Thinness (EDI-II)	17	(3)	3	(3)	224.000^b^	<0.001
Bulimia (EDI-II)	2	(2)	1	(2)	145.000^b^	0.187
Body Disatisfaction (EDI-II)	15	(5)	8	(6)	−3.576^a^	0.001
Perfectionism (EDI-II)	9	(4)	2	(2)	209.500^b^	<0.001
Interoceptive Awareness (EDI-II)	11	(7)	2	(3)	202.000^b^	<0.001
GM (ml)	714.40	(54.6)	732.74	(41.3)	−1.037^a^	0.308
WM (ml)	415.73	(50.8)	429.11	(39.2)	−0.083^a^	0.426
CSF (ml)	259.07	(49.7)	230.15	(31.3)	1.907^a^	0.067

*GM* gray matter, *WM* white matter, *CSF* cerebro-spinal fluid, *AN-r* anorexia nervosa-restrictive type, *HC* healthy control, *N* numbers, *SD* standard deviation, *BMI* body mass index

^a^Student’s *t*-test

^b^Mann–Whitney U test

### NBS analysis and VBM analysis

**Fig. 1:**
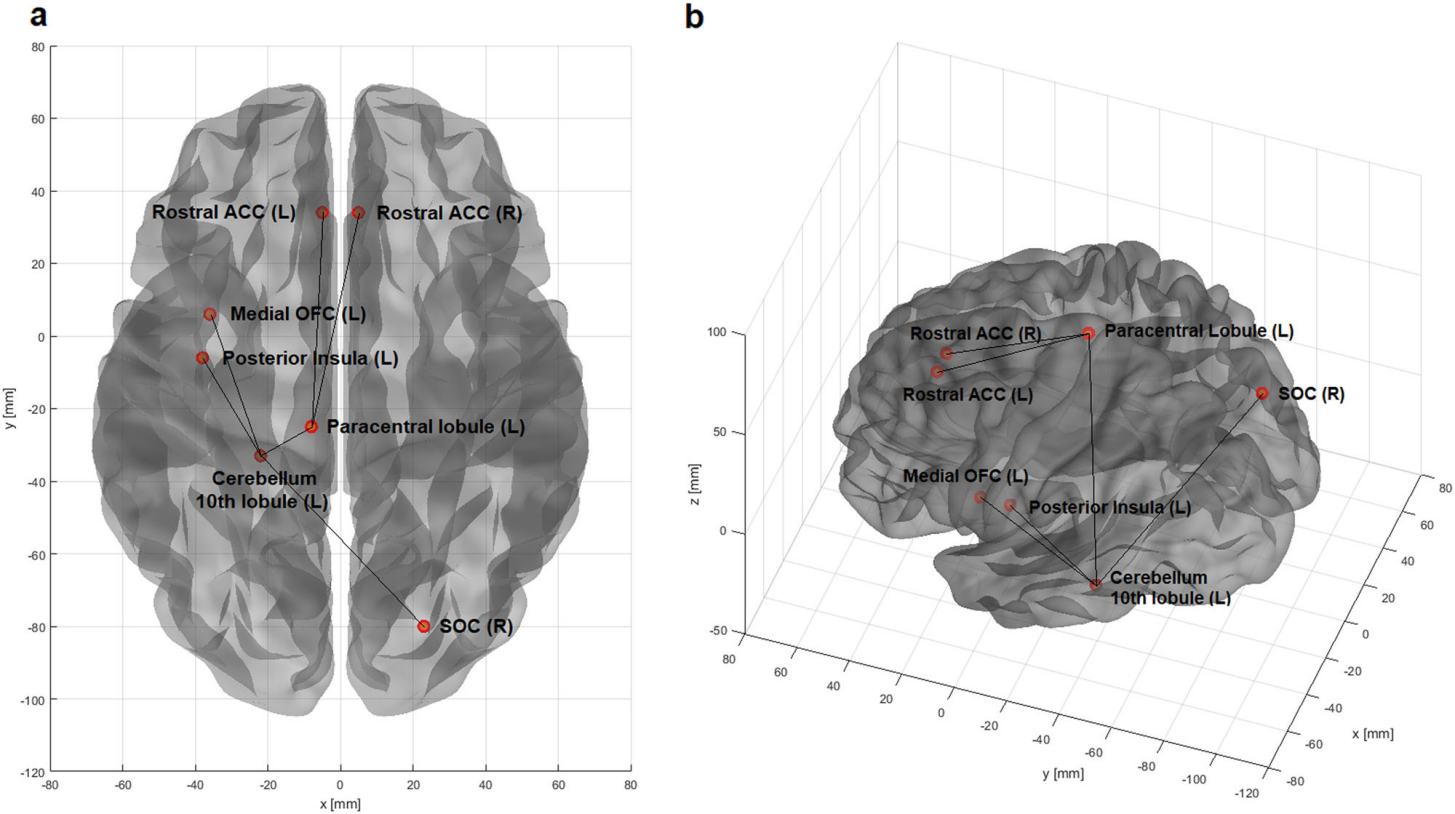
Network-based statistics results. The figure shows the sub-network with decreased connectivity in AN patients compared to controls, identified by the NBS. The nodes and the links are overlayed to a surface rendering of the brain in two different projections (**a**, axial; **b**, oblique). The brain surface with nodes representation was generated with the BRAPH toolbox

The NBS was performed with different primary thresholds, in the range between 2.0 and 4.0 (Table 2). The most significant difference between groups was observed at a threshold of *t* = 3.7, in the HC > AN contrast (*p* < 0.014) (Table 2). The resulting network comprised seven nodes (Fig. 1): left and right rostral ACC, left paracentral lobule, left cerebellum (10th lobule), left posterior insula, left medial orbitofrontal cortex (OFC), and right superior occipital cortex (SOC). Six connections were identified (Table 3): left medial OFC to left cerebellum, right SOC to left cerebellum, left paracentral lobule to left cerebellum, left cerebellum to left posterior insula, left paracentral lobule to left rostral ACC, left paracentral lobule to right rostral ACC. No differences were detected in the opposite contrast.

**Table 2:** Network-based statistics results at different primary thresholds (HC > AN)

Threshold	Nodes	Edges	*p*
2.0	127	531	0.042
2.1	127	441	0.044
2.2	122	367	0.043
2.3	119	297	0.047
2.4	–	–	ns
2.5	109	202	0.041
2.6	105	163	0.044
2.7	–	–	ns
2.8	–	–	ns
2.9	61	68	0.046
3.0	–	–	ns
3.1	30	31	0.043
3.2	21	21	0.036
3.3	–	–	ns
3.4	–	–	ns
3.5	7	6	0.047
3.6	7	6	0.027
3.7	7	6	0.014
3.8	5	4	0.029
3.9	5	4	0.017
4.0	–	–	ns

*ns* not significant

**Table 3:** Connections identified at the network-based statistics analysis

Node A	Node B
Left medial OFG	Left cerebellum (10th lobule)
Right SOC	Left cerebellum (10th lobule)
Left paracentral lobule	Left cerebellum (10th lobule)
Left cerebellum (10th lobule)	Left posterior insula
Left paracentral lobule	Left rostral ACC
Left paracentral lobule	Right rostral ACC

*Note*: All connections are undirected

VBM analysis, showed no significant GM differences between the AN outpatients and healthy controls. Inter-group analysis showed no significant differences in GM, WM, and CSF global volumes (see Table 1).

### Intranodal homogeneity and graph analysis

The KCC of the seven nodes identified by the NBS did not differ between groups. No correlations were found between the connectivity strengths and the KCC of the corresponding nodes, either in the patients or controls.

Regarding graph analysis, inter-group analysis showed no significant differences in global and local measures.

### Clinical-imaging correlations

No significant correlations were found in the clinical sample and in the control group separately between the selected clinical variables (i.e., BMI, BDI, STAI-trait, drive for thinness, body dissatisfaction, interoceptive awareness, perfectionism, bulimia, and AN duration) and the correlation coefficients relative to the connections identified by the NBS.

## Discussion

Ours is, to the best of our knowledge, the first study to explore RSFC, via a graph analysis approach (i.e., the NBS), in a sample of adolescents at the earliest stages of AN (i.e., AN duration less than 6 months) and age-matched healthy controls. Our AN sample showed a sub-network of lower connectivity encompassing bilateral rostral ACC, left paracentral lobule, left cerebellum (10th lobule), left posterior insula, left medial OFC, and right SOC compared to controls. AN patients did not show sub-network with a greater connectivity compared to controls.

Our results are only in a limited manner consistent with the previous NBS studies on AN patients^26^. In particular, Ehrlich and colleagues^26^ found a sub-network with a decreased connectivity mainly involving posterior insula and putamen in AN patients. In line with our findings, the sub-network alterations were not driven by intranodal homogeneity alterations^26^. Our results are only partially similar to the other three graph analysis studies on AN^27,28,47^. These three studies showed connectivity alterations of the insula, even if with different connections^27,28,47^. Connectivity alterations of the right posterior occipital cortex^27,28^ and bilateral OFC^47^ were also found. On the other hand, our findings are partially consistent with some of the previous resting-state studies which adopted a seed-based and ICA-based approach^23,48^. In particular, connectivity alterations within the cerebellar network, with an increased connectivity with insula and vermis, and a decreased connectivity with the parietal lobe were found^48^. Furthermore, altered functional connectivity of the ACC was found adopting a seed-based approach^23^.

The differences between our results and those of the previous NBS study^26^ can be largely related to differences in sample compositions. The previous recruited sample was composed by AN patients with a wide age range (12–23 years old) and also comprised patients with psychiatric comorbidity and AN binge-purging subtype^26^. Considering the three other graph analysis studies^27,28,47^, the differences between our results and those of these studies could be due to both the different composition of the samples and different resting-state approaches. It is of interest to note that the sample of the previous NBS study is the same^26^ as two other graph analysis studies and that different resting-state approaches gave partially different results^26–28^. Similarly, the differences between our results and those of ICA-based and seed-based studies can be related to the different sample composition (e.g., patients with long AN duration, psychiatric comorbidity, and previous or current pharmacological treatment) and the different resting-state approaches which only showed relatively consistent overlap in results^8,48^. On the whole, it can be suggested that the specific characteristics of our sample (i.e., subjects at the earliest stage of AN with no confounding factors) and the different resting-state approaches may mainly explain the differences between our results and the others. In particular, taking into account that partially different results can be found using different resting-state approaches^8^ and it also occurs studying the same sample^26–28^, our findings seem to suggest that functional connectivity may change during the course of AN. It is worth to note that only ACC alterations, as part of the executive control network, have been found in our previous whole-brain ICA study with a mostly overlapping sample^20^, suggesting that similar brain regions may be affected in the earliest stage of AN and leading to confirm that different resting-state approaches may yield partially different results also studying the same sample^26–28^.

The altered sub-network comprises brain areas distributed throughout the brain and involved in several functions. The lobule 10th of the cerebellum, also known as flocculonodular lobe, is mainly anatomically connected with sensorimotor cortices and vestibular nuclei, seems to be functionally activated by sensorimotor tasks, and the main effect of its damage is postural instability^49^. In particular, left cerebellar lesions seem to be related to deficits in visual-spatial performances^49^. Interestingly, it has been also suggested that the cerebellum may have a role in the development of the self, contributing to the construction of the subjective self which also includes homeostatic information and emotions^50^. The insula is connected to many brain areas and it is engaged in several functions and has a role in the processing of vestibular function, sensorimotor integration, integration of exteroceptive and interoceptive bodily signals and emotion^51,52^. In particular, the posterior insula seems to be mainly involved in general recognition of perceptual stimuli and skeleto-motor orientation^53^. The paracentral lobule is part of the sensorimotor cortex, functionally connected to different brain areas, and it is also involved in emotional and pain processing^54^. The rostral ACC has a complex anatomical connectivity and seems to play a key role in emotional, cognitive, and behavioral processes and in resolution of emotional conflicts^55,56^. In particular, it has been suggested that the rostral ACC may be involved, through interactions with insula and striatal regions, in integration of interoceptive, emotional, and cognitive functions^56^. Furthermore, the rostral ACC and paracentral lobule seem to be densely connected regions belonging to a structural core of human cerebral cortex which seems to play an important role in brain functional integration^57^. It is of interest to note that the right superior occipital gyrus, which is placed in the secondary visual cortex within the dorsal visual stream, is also involved in the mirror-induced visual illusion having influence on posterior parietal cortex and processing of visual information into motor commands^58^. Overall, the altered sub-network comprises brain areas with several functional connectivity and that are mainly involved in processing and integration of sensorimotor, interoceptive, and visual signals, as well as in emotional and cognitive processes.

Regarding our results, as a first hypothesis, it could be suggested that the functional connectivity alterations found in our sample could be related to AN symptomatology: underweight and altered nutritional state. Namely, the sub-network alterations may be an early non-specific functional connectivity abnormality related to AN symptomatology. However, no significant correlations were found between functional connectivity alterations and clinical variables (i.e., BMI and AN duration). These results seem to exclude that the functional alterations may be related to weight loss or duration of malnutrition. Moreover, our sample at the earliest stage of AN did not show significant GM differences, which may affect functional connectivity analyses^37,59^. In particular, considering that both global and regional GM atrophy can affect resting-state analyses and nodes connectivity^37^, this possible confounding effect seems to be excluded by the lack of detectable GM differences between groups. Therefore, it may be suggested a limited impact of malnutrition on our NBS findings.

Considering these last data, it seems rational to suggest, as a second hypothesis, that the altered cerebellar–insular–parietal–cingulate sub-network could be an early specific functional alteration directly related to the earliest stages of AN, despite the fact that no correlations were found between the functional connectivity alterations and self-reported symptom scores. Interestingly, no correlations were also found between functional alterations and clinical variables in the previous NBS study on AN^26^. This second interpretation seems to be supported by the observation that the altered sub-network encompasses brain areas also involved in somatosensory and interoceptive information and processing, as well as in emotional processes. This evidence seems to suggest that the altered sub-network could be related to complex body image disturbances of AN. Namely, it can be hypostasized that the altered sub-network may sustain an altered self-body image through an impaired processing and integration of somatosensory, interoceptive, and visual signals^8,26,60,61^, also involving emotional and cognitive processes^7,8^. From this point of view, it can be suggested that there could be a specific vulnerability of the altered sub-network areas/connections that could reflect an early bio-marker of AN and may have a role in AN pathophysiology. In addition, our previous results on mostly overlapping samples showed functional alterations between the executive control network and the ACC^20^ and microstructural alterations of WM tracts which connect the ACC to other regions (i.e., corona radiata)^29^ leading to confirm an involvement of the ACC in the earliest stages of AN.

### Limitations and strengths

The present study has some limitations that should be taken into consideration, as well as some methodological advantages. The first limitation is the limited sample size. This is mainly due to the strict inclusion criteria as well as to the low incidence of the disorder. The second limitation of the study is its cross-sectional design, which did not allow a longitudinal evaluation of the connectivity alterations over time. However, our aim was to investigate a sample of AN-r at the earliest stages of the disorder. Regarding this, the main strength of the study is the homogeneity of the sample. In fact, the clinical sample is composed of adolescents with AN-r at the earliest stages of the disease (i.e., AN-r in progress for less than 6 months at the time of scanning). Furthermore, patients did not have possible confounding factors such as previous or current psychopharmacological treatment^62^, or psychiatric comorbidity^63^. Although the exclusion of patients with psychiatric comorbidity limits possible confounding effects on neuroimaging analyses, a third limitation of the study is that the above-mentioned exclusion criterion may limit generalization of findings due to the well-known high rates of psychiatric comorbidity in AN.

## Conclusions

In conclusion, our adolescent sample of patients with AN at the earliest stages of the disease showed a reduced functional connectivity in a sub-network which comprises the rostral ACC bilaterally, left paracentral lobule, left cerebellum (10th lobule), left posterior insula, left medial OFC, and right SOC compared to controls. Our results suggest that functional connectivity at rest may be specifically affected since the earliest stages of AN. The brain areas of the altered sub-network are mainly involved in processing of somatosensory and interoceptive signals and in emotional processes, leading to suggest that they may have a role in AN pathophysiology and could be involved and sustain body image disturbances in AN. Further studies with larger samples and longitudinal designs, which may explore functional changes over time and consider relationship between functional connectivity alterations and AN brain atrophy, are needed to confirm our results and to clarify the role of such functional alterations.

## Electronic supplementary material

Supplementary material
