# Supplementary material for: Altered cerebellar–insular–parietal–cingular subnetwork in adolescents in the earliest stages of anorexia nervosa: a network–based statistic analysis
Source: Transl Psychiatry. 2018 Jul 6;8:127. doi: 10.1038/s41398-018-0173-z (PMC6035187; doi:10.1038/s41398-018-0173-z)
Supplement: Supplementary file 1 — Supplementary material [file 41398_2018_173_MOESM1_ESM.rtf]

Altered cerebellar-insular-parietal-cingular subnetwork in adolescents in the earliest stages of anorexia nervosa: a network based statistic analysis

Graph analysis: global and local measures
Local measures. Seven local measures were computed: strength, global efficiency of the nodes (ENg), local efficiency of the nodes (ENl), clustering coefficient of the nodes (CCn), betweenness centrality (BC) and participation coefficient (PC). The strength of a node is the sum of the weights of the edges connected to the node. The ENg is the average inverse shortest path length of the node. The ENl is the global efficiency of the node computed on the node's neighbourhood. The CCn represents the fraction of a node's neighbors that are neighbors to each other; the CCn is a measure of segregation, and reflects the ability for specialized processing in small groups of nodes, i.e. the belonging of the node to a local subnetwork. BC of a node is the fraction of all shortest paths in the graph that contain a given node; nodes with high values of BC participate in a large number of shortest paths (subnetworks). The PC assesses the diversity of intermodular interconnections of individual nodes; nodes with a high within-module degree but with a low PC (known as provincial hubs) are hence likely to play an important part in the facilitation of modular segregation. On the other hand, nodes with a high PC (known as connector hubs) are likely to facilitate global intermodular integration.
Global measures. We computed six global measures: characteristic path length (CPL), global efficiency (Eg), local efficiency (El), clustering coefficient (CC), assortativity and  small-worldness. The CPL is a measure of the efficiency of information transfer in a network, and represents the average minimum number of edges that must be traversed to go from one node to another. The Eg is the average inverse shortest path length in the graph, and is inversely related to the characteristic path length. The El is the average of the local efficiencies of its nodes, and reflects how well the nodes communicate with adjacent nodes. The CC is the average of the clustering coefficients of its nodes. The assortativity coefficient is a correlation coefficient between the degrees/strengths of all nodes on two opposite ends of a link; a positive assortativity coefficient indicates that nodes tend to link to other nodes with the same or similar degree/strength.


Table S1. List of ROIs.
ROI Labels		MNI coordinates		
aal	Side	x	y	z	Side	x	y	z	
Precentral Gyrus	L	-40	-6	51	R	40	-8	52	
Superior Frontal Gyrus	L	-19	35	42	R	21	31	44	
Superior Orbitorfrontal Gyrus	L	-18	47	-13	R	17	48	-14	
Middle Frontal Gyrus	L	-34	33	35	R	37	33	34	
Middle Orbito-frontal Gyrus	L	-32	50	-10	R	32	53	-11	
Inferior Frontal Gyrus (p. opercularis)	L	-49	13	19	R	49	15	21	
Inferior Frontal Gyrus (p. triangularis)	L	-47	30	14	R	49	30	14	
Inferior Orbito-frontal Gyrus	L	-37	31	-12	R	40	32	-12	
Rolandic Operculum	L	-48	-8	14	R	52	-6	15	
Supplementary Motor Area	L	-6	5	61	R	8	0	62	
Olfactory Gyrus	L	-6	54	-7	R	7	52	-7	
Superior Medial Frontal Gyrus	L	-6	37	-18	R	7	36	-18	
Medial Orbito-frontal Gyrus	L	-36	7	3	R	38	6	2	
Rectus Gyrus	L	-5	35	14	R	7	37	16	
Middle Cingulate Cortex	L	-6	-15	42	R	7	-9	40	
Posterior Cingulate Cortex	L	-6	-43	25	R	6	-42	22	
Hippocampus	L	-26	-21	-10	R	28	-20	-10	
ParaHippocampal Gyrus	L	-22	-16	-21	R	24	-15	-20	
Amygdala	L	-24	-1	-17	R	26	1	18	
Calcarine Cortex	L	-8	-79	6	R	15	-73	9	
Cuneus	L	-7	-80	27	R	13	-79	28	
Lingual Gyrus	L	-16	-68	-5	R	15	-67	-4	
Superior Occipital Gyrus	L	-18	-84	28	R	23	-81	31	
Middle Occipital Gyrus	L	-33	-81	16	R	36	-80	19	
Inferior Occipital Gyrus	L	-37	-78	-8	R	37	-82	-8	
Fusiform Gyrus	L	-32	-40	-20	R	33	-39	-20	
Postcentral Gyrus	L	-43	-23	49	R	40	-25	53	
Superior Parietal Gyrus	L	-24	-60	59	R	25	-59	62	
Inferior Parietal Gyrus	L	-44	-46	47	R	45	-46	50	
Supramarginal Gyrus	L	-57	-34	30	R	57	-32	34	
Angular Gyrus	L	-45	-61	36	R	45	-60	39	
Precuneus	L	-8	-56	48	R	9	-56	44	
Paracentral Lobule	L	-9	-25	70	R	6	-32	68	
Caudate Nucleus	L	-12	11	9	R	14	12	9	
Putamen	L	-25	4	2	R	27	5	2	
Pallidum	L	-19	0	0	R	20	0	0	
Thalamus	L	-12	-18	8	R	12	-18	8	
Heschl's Gyrus	L	-43	-19	10	R	45	-17	10	
Superior Temporal Gyrus	L	-54	-21	7	R	57	-22	7	
Superior Temporal Pole	L	-41	15	-20	R	47	15	-17	
Middle Temporal Gyrus	L	-57	-34	-2	R	56	-37	-1	
Middle Temporal Pole	L	-37	15	-34	R	43	15	-32	
Inferior Temporal Gyrus	L	-51	-28	-23	R	53	-31	-22	
Cerebellum Crus1	L	-36	-67	-29	R	37	-67	-30	
Cerebellum Crus2	L	-29	-73	-38	R	32	-69	-40	
Cerebellum 3	L	-9	-37	-19	R	12	-34	-19	
Cerebellum 4/5	L	-15	-43	-17	R	17	-43	-18	
Cerebellum 6	L	-23	-59	-22	R	25	-58	-24	
Cerebellum 7b	L	-32	-60	-45	R	33	-63	-48	
Cerebellum 8	L	-26	-55	-48	R	25	-56	-49	
Cerebellum 9	L	-11	-49	-46	R	9	-49	-46	
Cerebellum 10	L	-23	-34	-42	R	26	-34	-41	
Vermis 1/2	-	1	-39	-20					
Vermis 3	-	1	-40	-11					
Vermis 4/5	-	1	-52	-6					
Vermis 6	-	1	-67	-15					
Vermis 7	-	1	-72	-25					
Vermis 8	-	1	-64	-34					
Vermis 9	-	1	-55	-35					
Vermis 10	-	0	-46	-32					
Deen, 2011									
Insula (ventral-anterior)	L	-33	13	-7	R	32	10	-6	
Insula (postero-anterior)	L	-38	6	2	R	35	7	3	
Insula (posterior)	L	-38	-6	5	R	35	-11	6	
Yun, 2017; Zhou, 2016									
Anterior Cingulate Cortex (caudal)	L	-5	-10	47	R	5	-10	47	
Anterior Cingulate Cortex (dorsal)	L	-5	14	42	R	5	14	42	
Anterior Cingulate Cortex (rostral)	L	-5	34	28	R	5	34	28	
Anterior Cingulate Cortex (perigenual)	L	-5	47	11	R	5	47	11	
Anterior Cingulate Cortex (subgenual)	L	-5	25	-10	R	5	25	-10	
